# Mawangdui-Guidance Qigong Exercise for patients with chronic non-specific low back pain: Study protocol of a randomized controlled trial

**DOI:** 10.3389/fnins.2023.1090138

**Published:** 2023-03-13

**Authors:** Guilong Zhang, Liang Gao, Di Zhang, Hongjian Li, Yuquan Shen, Zhengsong Zhang, Yong Huang

**Affiliations:** ^1^Department of Orthopedics, Hospital of Chengdu University of Traditional Chinese Medicine, Chengdu, China; ^2^Beijing Bo’ai Hospital China Rehabilitation Research Center, School of Rehabilitation, Capital Medical University, Beijing, China; ^3^Department of Rehabilitation, Hospital of Chengdu University of Traditional Chinese Medicine, Chengdu, China; ^4^Department of Orthopedics, Yibin Hospital of Traditional Chinese Medicine, Yibin, Sichuan, China; ^5^Department of Rehabilitation, The First People’s Hospital of Longquanyi District, Chengdu, China; ^6^Traditional Chinese Medicine (TCM) Preventive Medical Center, Hospital of Chengdu University of Traditional Chinese Medicine, Chengdu, China

**Keywords:** Qigong, postural balance, paraspinal muscles, RCT, low back pain

## Abstract

**Introduction:**

Worldwide, there is a high frequency of chronic non-specific low back pain (CNLBP), which is a significant public health concern. The etiology is complicated and diverse, and it includes a number of risk factors such as diminished stability and weak core muscles. Mawangdui-Guidance Qigong has been employed extensively to bolster the body in China for countless years. However, the effectiveness of treating CNLBP has not been assessed by a randomized controlled trial (RCT). In order to verify the results of the Mawangdui-Guidance Qigong Exercise and examine its biomechanical mechanism, we intend to perform a randomized controlled trial.

**Methods and analysis:**

Over the course of 4 weeks, 84 individuals with CNLBP will be randomly assigned to receive either Mawangdui-Guidance Qigong Exercise, motor control exercise, or medication (celecoxib). Electromyographic data, including muscle activation time, iEMGs, root mean square value (RMS) and median frequency (MF), will be the main outcomes. The Japanese Orthopedic Association (JOA) Score, the Mcgill Pain Questionnaire (MPQ), beta-endorphin, and substance P are examples of secondary outcomes. At the start of treatment and 4 weeks later, all outcomes will be evaluated. SPSS version 20.0 (SPSS Inc., Chicago, IL, USA) will be used for all of the analysis.

**Discussion:**

The prospective findings are anticipated to offer an alternative treatment for CNLBP and provide a possible explanation of the mechanism of Mawangdui-Guidance Qigong Exercise on CNLBP.

**Ethics and dissemination:**

The Sichuan Regional Ethics Review Committee on Traditional Chinese Medicine has given the study approval (Approval No. 2020KL-067). It has also registered at the website of China Clinical Trial Center Registration. The application adheres to the Declaration of Helsinki’s tenets (Version Edinburgh 2000). Peer-reviewed papers will be used to publicize the trial’s findings.

**Trial registration number:**

ClinicalTrials.gov, identifier ChiCTR2000041080.

## Strengths and weaknesses of this research

1. The purpose of this study protocol is to conduct the first-ever single-blinded, randomized controlled trial to assess the effectiveness and potential mechanism of Mawangdui-Guidance Qigong Exercise for CNLBP.
2. Biological markers and EMG data are included in the primary and secondary outcomes. There has been no agreement achieved despite numerous EMG investigations that attempted to disclose the CNLBP with muscle activity sequence mechanism. Beta-endorphin levels and substance P, which are quick, safe, and effective markers to gauge pain relief will be tested.
3. Participants are limited by ages between 20 and 39 for easily identification of specific chronic LBP or CNLBP, further researches on differences of various ages are needed.
4. This is a single-center RCT with limited sample size, multi-center experiments can be conducted to get reproducible and stable results in the future.
5. The treatment duration keeps 4 weeks without longer-term observation, we plan to conduct a follow-up at the 8th and 12th weeks by phone after the trial finished.

## Introduction

Pain between the costal margin and inferior gluteal fold is known as low back pain (LBP). Chronic non-specific low back pain (CNLBP) is defined as LBP for at least 12 weeks, which is not caused by a specific pathology such as a tumor, infection, fracture, structural deformity, radiculopathy, osteoporosis, inflammatory disorder, or cauda equina syndrome (Furlan et al., 2009). The literature reports a wide range of estimated prevalence rates for CNLBP, ranging from 4 to 14% (Parthan et al., 2006; van Tulder et al., 2006; Chou et al., 2007). One of the most prevalent musculoskeletal conditions in the world today is CNLBP (Maher et al., 2017). Effective therapies from drugs to surgery for CNLBP are widely used (Ma et al., 2019). NSAIDs, such as Celecoxib and other COX-2 inhibitors are frequently advised for the treatment of CNLBP (Birbara et al., 2003; Katz et al., 2003; Pallay et al., 2004; Airaksinen et al., 2006). The effectiveness of exercise therapy in CNLBP, including pilates, Tai Chi, yoga, and motor control exercise (MCE), has received greater attention in recent years (van Middelkoop et al., 2011; Holtzman and Beggs, 2013; Macedo et al., 2013; Eliks et al., 2019). For the treatment of persistent, nonspecific LBP, exercise therapy has been utilized extensively (Saragiotto et al., 2016).

Mawangdui-Guidance Qigong Exercise is an arising exercise therapy organized and created by the Fitness Qigong Management Center of the State General Administration of Sport of China (Fan, 2002). Traditional Chinese exercises, including Qigong and Tai Chi, are recommended to relieve pain intensity in patients with LBP (Zhang et al., 2019). Qigong may achieve the same efficacy as other exercise therapies in the treatment of CNLBP (Blödt et al., 2015). The pain intensity and back dysfunction are significantly regulated for people who practice Qigong (Phattharasupharerk et al., 2019), and it is safe in the treatment of musculoskeletal pain (Marks, 2019).

In order to assess internal and external postural interference in patients with LBP, the quick arm raise test and the falling ball test by surface electromyograph (sEMG) are frequently utilized (Hodges and Richardson, 1996; Tsao and Hodges, 2008; Jacobs et al., 2017; Xie and Wang, 2019; Larivière and Preuss, 2021; Yu et al., 2021). Although no consensus was established, numerous EMG studies attempted to shed light on the CNLBP with muscle activity sequence mechanism (Geisser et al., 2005; Marshall and Murphy, 2008; Falla and Hodges, 2017). The influence of adjusting posture on pain reduction and disability improvement may be observed by using EMG to examine the changes in muscle initiation time and iEMGs in patients with CNLBP (Yu et al., 2021). The management of spinal postural alignment and overall body balance are thought to depend mostly on the back muscles (Daggfeldt and Thorstensson, 2003; Christophy et al., 2012). The Chinese Association for the Study of Pain (Ma et al., 2019) advises sEMG y as an objective method of assessing the back muscles’ functionality (Akbari et al., 2015).

There is not enough solid evidence to support the high efficacy of Qigong when compared to other various nonoperative treatments. Mawangdui-Guidance Qigong Exercise has not been subjected to a randomized controlled trial (RCT) to determine the effectiveness in treating CNLBP. We strive to confirm the critical function in treating chronic CNLBP by contrasting Mawangdui-Guidance Qigong Exercise with MCE and medication.

## Methods and design

### Study design

This study is a randomized, single-blinded clinical trial to investigate the effectiveness of Mawangdui-Guidance Qigong Exercise for CNLBP. This study will be conducted at the Hospital of Chengdu University of Traditional Chinese Medicine (CDUTCM). For the duration of the 4-week course of therapy, 84 participants who meet the trial criteria will be assigned at random to the Mawangdui-Guidance Qigong Exercise group, the MCE group, or the medicine group. Participants in Qigong group and MCE group will receive different types of exercise therapies under the supervision of different professional coaches. Others in the medicine group will be given celecoxib orally on a regular basis. EMG data, including muscle activation time, iEMGs, root mean square value (RMS) and median frequency (MF), will be the main outcomes. The Japanese Orthopedic Association (JOA) Score, the Mcgill Pain Questionnaire (MPQ), beta-endorphin, and substance P will be the secondary outcomes. All outcomes will be evaluated at the start of treatment and 4 weeks later. We think Mawangdui-Guidance Qigong Exercise can produce an improvement in pain and lumbar function that is equal to or greater than what the other two groups can.

**FIGURE 1:**
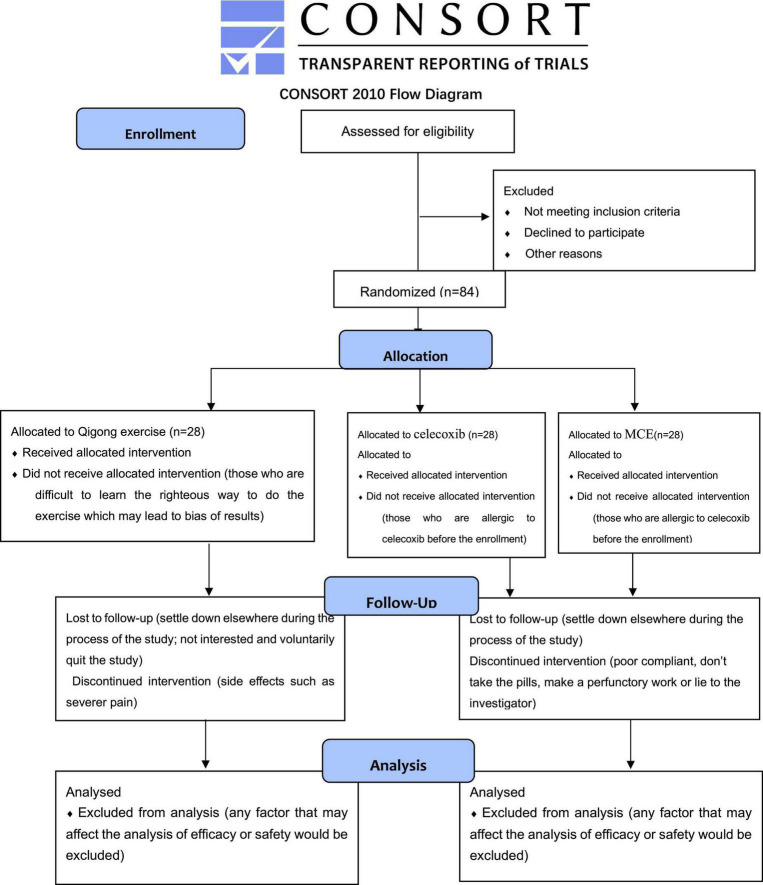
Flow chart of the whole study design.

The study is approved by the Sichuan Regional Ethics Review Committee on Traditional Chinese Medicine (Approval No. 2020KL-067) and registered with China Clinical Trial Center Registration (ChiCTR2000041080). The implementation follows the principles of the Declaration of Helsinki (Version Edinburgh 2000). The SPIRIT guidelines and CONSORT flow diagram will be followed in this investigation. The whole study design will be illustrated as a flow chart in Figure 1 and the process timetable will be shown in Table 1.

**TABLE 1:** Process timetable.

		Study period							
		Enrollment	Allocation	Post allocation			Close-out	Follow-up	
Timepoint	Week	−1	0	1	2	3	4	8	12
**Enrollment**									
Inclusion/exclusion criteria		√							
Informed consent		√							
Medical history		√		√	√	√	√		
Laboratory tests		√					√		
Muscle action time, EMG data		√					√		
JOA, MPQ		√					√		
Random allocation			√						
**Interventions**									
Mawangdui-Guidance Qigong Exercise				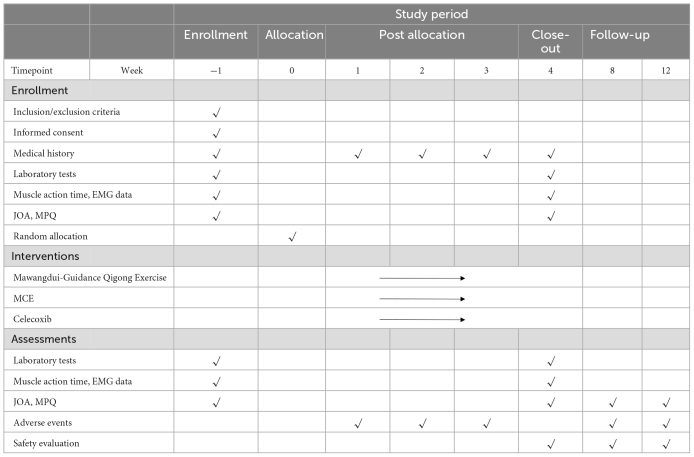					
MCE				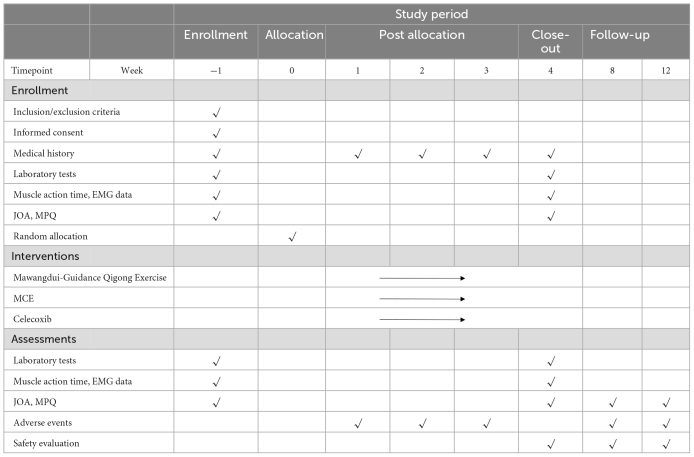					
Celecoxib				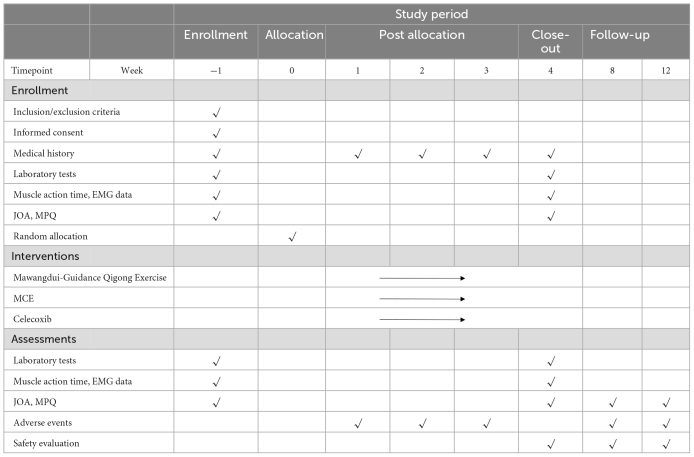					
**Assessments**									
Laboratory tests		√					√		
Muscle action time, EMG data		√					√		
JOA, MPQ		√					√	√	√
Adverse events				√	√	√		√	√
Safety evaluation							√	√	√

### Inclusion criteria

Eligible participants who met the following criteria will be included: (i) pain between the costal margin and inferior gluteal fold for at least 12 weeks which is not caused by a specific pathology, (ii) age between 20 and 39 years old, (iii) never received drug treatment, non-pharmacy, or surgical treatments for CNLBP during the previous 12 weeks, and (iv) agree to join the study and sign the written informed consent.

### Exclusion criteria

Participants matching any of the following criteria will be excluded: (i) LBP with a specific diagnosis such as lumbar disc herniation, fracture of lumbar vertebra, spinal stenosis, severe osteoarthritis, or ankylosing spondylitis, (ii) be suffering from serious diseases of the heart, liver, kidney, or other organs, (iii) pregnant women, tumor patients, or those with serious disorders, (iv) structural deformity of the lumbar spine, (v) be suffering from other diseases that cause pain, such as migraine, angina, etc., and (vi) patients received surgery, acupuncture, massage, Tuina, spinal manipulation, and NSAIDs treatment in the past 12 weeks.

### Interventions

#### Mawangdui-Guidance Qigong Exercise therapy

**FIGURE 2:**
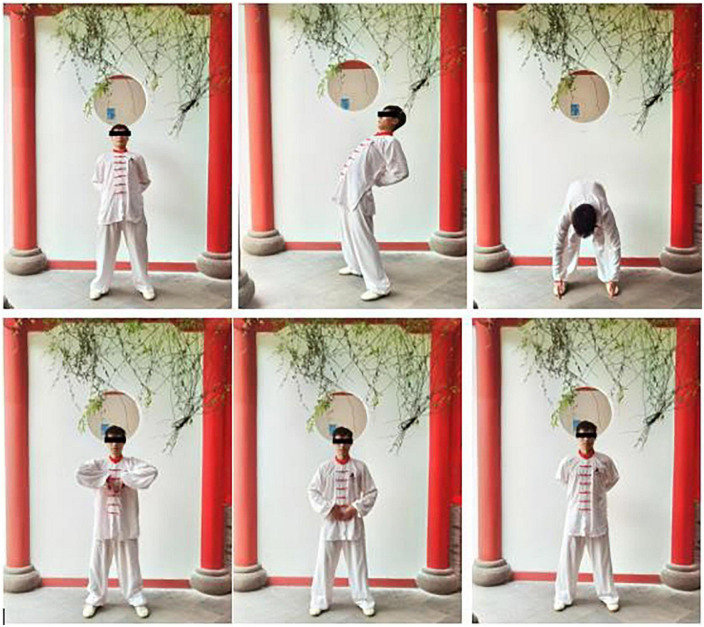
The action of the Mawangdui-Guidance Qigong Exercise’s “yinyao” movement.

For a session of 4 weeks, participants in the experimental group will receive Mawangdui-Guidance Qigong Exercise therapy five times each week. Each exercise will continue for 30 min and be led by a particular coach who is equally skilled and experienced and has received training in how to manage a project. Figure 2, Table 2, and Supplementary video 1 show the program of the Mawangdui-Guidance Qigong Exercise’s “yinyao” movement.

**TABLE 2:** The steps of the Mawangdui-Guidance Qigong Exercise’s “yinyao” movement training.

i	Relaxed standing, both hands staying at palm, move both palms from abdomen to waist along Belt Vessel with hands sticking to it; palms brace against waist and push it ahead to the body leaned forward.
ii	Both palms move downward to hip and turn body bended, keep moving palms along the back of thighs and shins to heel and moving palms from heel to forward, hanging the arms and palms upon the tiptoes, rise head and look straight ahead.
iii	With the last posture referred in (ii), rotate the waist and lift the left shoulder and palm at the same time, meanwhile, turn the head backward and look to left rear.
iv	Rotate the waist and droop the left shoulder, meanwhile, turn back the head and look to infra-anterior.
v	Lift the body upright as well as the palms lift to the chest with it rotating interior and back of the palms being against to each other along the anterior median line, and look straight ahead.
vi	Move both palms to belly, and move them from abdomen to waist along Belt Vessel with hands sticking to it; palms brace against waist and push it ahead to the body leaned forward.

The main movement is the same as ii–v, except for the rotation of head, waist, and shoulder turns to right. Both palms fall at both sides until the second movement terminated and look straight forward.

#### Motor control exercise

The main goal of these exercises in the MCE group is to normalize the significant motor abnormalities by teaching participants to maintain stable posture while properly contracting their muscles to lessen pain and intensity. Precious study helps to set the MCE strategy (Li et al., 2021). Participants will practice five times a week for 30 min each under the guidance of a specified experienced physiotherapist. Table 3 depicts the MCE process steps.

**TABLE 3:** The steps of motor control exercise.

i	In the first step, the participants’ ability to control the joint neutral position in the four-point kneeling position will be retrained. The participants learned how to find their postural balance in the basic training.
ii	In the second step, the participants will learn how to control movements in their lumbar spine with minimal effort while moving their arms and legs. Participants will be asked to lift one limb to a horizontal position for 5 s during four-point kneeling. Each limb will be repeated thrice with a break of 15 s.
iii	In the third step, the participants will lift one arm and the contralateral leg to a horizontal position for 5 s. This action will also be repeated three times with a break of 15 s.

#### Drug treatment

A qualified and experienced doctor who has received the same training as the medicine group will evaluated and give oral celecoxib (Celebrex, Pfizer), 200 mg once daily, for 4 weeks to participant. The doctor will provide drug counseling throughout the duration of the experiment, as well.

To guarantee impartiality and rigor, participants with increased muscle pain after exercise and no obvious relief after rest will be treated with appropriate cryotherapy under the supervision of researchers. However, additional treatments including decoction, Chinese herbs, acupuncture, Tuina, and other painkillers will not be permitted.

### Outcome measures

#### Primary outcomes

Primary outcomes include muscle activation sequence (Hodges, 2001; Suehiro et al., 2015), iEMG, RMS, and MF, generated from sEMG (Thomas et al., 2007; Aruin et al., 2015; Aruin, 2016). The EMG data will be gathered from the probe on the surface of deltoids, lumbar multifidus, transverse abdominis muscle (TrA), erector spinae muscle, gluteus maximus, and hamstrings. Each channel’s interelectrode distance will be 10 mm.

#### Secondary outcomes

The JOA Score (Oshima et al., 2020) and the McGill Pain Questionnaire (MPQ) (Main, 2016) are the secondary outcomes. JOA Score takes into account bladder function, daily activity, clinical indicators and subjective complaints, with the lowest score of 6 and the highest score of 29. Better functioning conditions are indicated by higher overall scores. MPQ is made up of 15 carefully chosen words, comprising 4 emotional and 11 sensory words. Each item is given a number from 0 to 3, with a higher score denotes a severer symptom (Choi et al., 2015). Biomarkers like substance P (Bhatia, 2015) and beta-endorphin (Choi and Lee, 2019) will also be tested.

All the primary and secondary outcomes will be assessed before and 1 week after treatment.

#### Adverse events

Researchers will keep track of adverse events and evaluate the correlation with therapies. Once relevant severe adverse event occurs (major damage to the heart, liver, kidneys, or other organs), the researchers will evaluate the participant can continue this trial or not. If serious adverse reactions occur, visits will continue after safety and treatment after the patient’s trial is suspended.

### Sample size

By utilizing a two-sided 0.05 level *t*-test with >95% power and assuming a 10% dropout rate, the sample size was estimated to demonstrate the influence of the target muscles on MF (Zhou, 2018). To detect a target effect size of 0.44 (GPower 3.1) with 28 participants in each group, a sample size of 84 participants is needed.

### Participants recruitment

Chronic non-specific low back pain is an exclusionary diagnosis. Patients with chronic LBP who do not have particular illnesses will be given this diagnosis (Koes et al., 2006). Eligible patients will be recruited from the Department of Orthopedics of Chengdu University of Traditional Chinese Medicine (CDUTCM). To ensure that recruitment messages are easily received by patients interested in the trial, recruitment posters and leaflets are placed in hospital lobbies and orthopedic clinics. Patients can also contact the staff through WeChat and the hospital website to sign up for the trial. In order to improve compliance, participants will be fully informed before signing informed consent. Researchers will keep track of participants’ compliance, including monitoring drug use and exercise on a regular basis. The participant recruiting process started on 23 October 2022, is still running, and it should be finished by 31 December 2022.

### Allocation

Eligible patients will be randomly assigned to the Qigong group (*n* = 28), the MCE group (*n* = 28), or the medication group (*n* = 28) according to the inclusion and exclusion criteria with a ratio of 1:1:1. Prior to intervention, each patient will receive a random number in a sealed envelope. An automated random number generator created this random number, which is specific to each row in the table. A randomizer will assign patients to the experimental or control groups. An assessor who is unrelated to the study will maintain absolute confidentiality regarding the allocation list.

### Blinding

In this study, a single blind approach will be used to execute the Mawangdui-Guidance Qigong Exercise. It is impossible to blind participants and researchers to the group assignment. The same unbiased, skilled evaluators who are blind to the allocation will measure each result.

### Data collecting and monitoring

The Data Monitoring Committee (DMC) for Medical Data in this project is the Chengdu University of Traditional Chinese Medicine Evidence-based Medicine Center. Designated outcome assessors will record data on paper and computerized CRFs, and the DMC will keep an eye on it. Every 3 months, monitors will audit the data. During the evaluation process, coaches and statisticians will not have access to these data.

### Statistical analysis

Test-retest reliability and content consistency reliability will be used to gauge the scale’s dependability (test-retest reliability). Cronbach’s coefficient is the most often used measure of internal consistency reliability. The better the homogeneity, the higher the Cronbach’s alpha coefficient. If the scale’s consistency is greater than 0.8, it is considered to be good, and if it is greater than 0.7, it is considered to be acceptable.

SPSS version 20.0 (SPSS Inc., Chicago, IL, USA) will be used for all of the analysis. The significance threshold will be set at 0.05, and the confidence interval will be 95%. The χ^2^ will be used to analyze categorical data. For continuous variables, mean ± SD will be recorded by using ANOVA test if they were normally distributed, or the median with interquartile range will be shown with Kruskal–Wallis H test. The Bonferroni correction and Tukey *post-hoc* test (for ANOVA) will be used to handle multiple comparisons (for Kruskal–Wallis). The Chi-square test will be used to verify the classification count data. Two-tailed test will be used in this study. The difference will be regarded as statistically significant when *P* < 0.05.

To handle missing data, we will evaluate the underlying cause, employ an imputation adjustment approach, and do a last observation carried forward analysis. Following the primary analysis, a sensitivity analysis will be carried out to determine the effect of missing data on the trial outcomes by contrasting the findings from the per-protocol analysis and the intention to treat analysis. Also planned are subgroup analysis by the center.

### Quality assurance

Before agreeing to participate, it will be ensured that each participant meets tight eligibility requirements. All of the researchers have received professional training to understand how to carry out a typical research protocol and the operational requirements. Particularly, there will be individuals in charge of gathering and registering test information. Periodically, the data will be audited by the DMC monitors.

## Discussion

The purpose of this study is to explore the effectiveness of Mawangdui-Guidance Qigong Exercise on CNLBP, and to further discuss the potential neuroelectrophysiology mechanism. Lumbar multifidus muscle has been proved crucial to the intersegmental stability of lumbar spine (Beimborn and Morrissey, 1988). Higher level of multifidus muscle activation observed in CNLBP patients is related to muscle spasm and the Pain Adaptation Model (Sherman, 1985; Ahern et al., 1988; Ansari et al., 2018). TrA primarily contributes to the dynamic stability of the lumbar spine by contracting the abdominal muscles to regulate various pain adaption models (van Dieën et al., 2003; Allison and Morris, 2008; da Silva et al., 2017). The data of lumbar multifidus muscle and TrA (Beneck et al., 2016; Shah et al., 2020) are usually collected using sEMG in the study of postural balance in patients with LBP. The relationship between neuromuscular control mechanisms and pain, however, is yet unclear.

Neurological system moderately activates the muscles at the proper time in the event of spinal injury, according to the “spinal stability model” (Panjabi, 1992). Feed-forward, feed-back, and voluntary control makes up of the basic central motor control modes of lumbar spinal stability muscles. According to visual, auditory, postural, and proprioceptive senses as well as feed-forward and feed-back control, the nervous system regulates the lumbar stability muscles to maintain lumbar stability or unconsciously protect the spine. The probable mechanism of Qigong may involve stretching and strengthening the core muscles in the waist. In patients with CNLBP, Mawangdui-Guidance Qigong Exercise, according to our hypothesis, preserves postural stability by altering the order of muscle activation.

Since the pathophysiology of CNLBP is unknown, LBP is frequently included in discussions about CNLBP illness management (Maher et al., 2017). Inflammation may continue throughout the entire course of LBP (Vucetic and Svensson, 1996; Vroomen et al., 2002) and is a key component of both pain and spinal degenerative processes (Wuertz and Haglund, 2013; Risbud and Shapiro, 2014). Beta-endorphin and substance P are also advised as alternatives to inflammatory markers as indicators of how well a treatment is working for patients with LBP (Bhatia, 2015; Choi and Lee, 2019). We believe that Mawangdui-Guidance Qigong Exercise can achieve the same or even more improvement in pain and lumbar function than the other two groups.

Prescription of Mawangdui-Guidance Qigong Exercise revolve limb opening and closing, rotation flexion and extension, stretching, and bone stretching, based on meridian guidance. It is a popular kind of Traditional Chinese Medicine (TCM) rehabilitation exercise therapy recently for cardiovascular, metabolic, and musculoskeletal system diseases (Wang et al., 2014; Sun and Wang, 2015; Chen, 2016; Ding et al., 2019). Though it is beneficial to the changes in the cellular level of elderly women (Wang et al., 2016), generally speaking, application of guidance still focuses on the observation of the overall effect on the body. The current research should focus on the therapeutic impact mechanism of Mawangdui guidance.

Compared with younger people, people over the age of 40 have a higher incidence of degenerative diseases such as lumbar disc herniation (Brinjikji et al., 2015). It is difficult to prove that LBP in patients over 40 years old is not caused by the above-mentioned specific causes. The specificity and non-specificity of LBP can be easily identified in younger people. That is why we designed this RCT with participants limited by ages between 20 and 39. As a result, the current study could not clearly explain the differentiation between various ages, and future researches on the effectiveness of multiple ages are needed. Secondly, since the single-center design with limited sample size may restrict the credibility of this trial, multi-center experiments in the future may help in enhancing the reproducibility and stability of the research. In addition, the treatment duration keeps 4 weeks without longer-term observation, we plan to conduct a follow-up at the 8th and 12th weeks by phone after the trial finished.

By contrasting the efficacy and safety of Mawangdui-Guidance Qigong Exercise in CNLBP treatment with MCE and oral medication, the prospective findings are anticipated to offer an alternative treatment for CNLBP and provide a possible explanation of the mechanism of Mawangdui-Guidance Qigong Exercise on CNLBP.

## Ethics statement

Written informed consent was obtained from the individual(s) for the publication of any potentially identifiable images or data included in this article.

## Author contributions

GZ, LG, YS, ZZ, and YH participated in the conception and design of this trial. GZ, DZ, and HL were responsible for planning the draft and revising the manuscript. GZ was monitor of this study. All authors contributed to this work, read the manuscript, and approved the publication of this protocol.
